# Sequential Continuous Mixotrophic and Phototrophic Cultivation Might Be a Cost-Effective Strategy for Astaxanthin Production From the Microalga *Haematococcus lacustris*

**DOI:** 10.3389/fbioe.2021.740533

**Published:** 2021-10-05

**Authors:** Mahammed Ilyas Khazi, Liangtao Shi, Fakhra Liaqat, Yuxin Yang, Xin Li, Duanpeng Yang, Jian Li

**Affiliations:** ^1^ Department of Research and Development, Panzhihua Gesala Biotechnology Inc., Panzhihua, China; ^2^ Institute of Tropical Eco-agriculture, Yunnan Academy of Agricultural Science, Kunming, China; ^3^ School of Biological and Chemical Engineering, Panzhihua University, Panzhihua, China

**Keywords:** astaxanthin, *Haematococcus*, mixotrophy, microalgae, continuous cultivation

## Abstract

Although *Haematococcus lacustris* has been developed for astaxanthin production for decades, the production cost is still high. In order to modify the production processes, we proposed a novel strategy of cultivation, featured by sequential indoor continuous mixotrophic cultivation for the production of green cells followed by outdoor phototrophic induction for astaxanthin accumulation. The continuous mixotrophic cultivation was first optimized indoor, and then the seed culture of mixotrophic cultivation was inoculated into outdoor open raceway ponds for photoinduction. The results showed that mixotrophically grown cultures could efficiently grow without losing their photosynthetic efficiency and yielded higher biomass concentration (0.655 g L^−1^) and astaxanthin content (2.2% DW), compared to phototrophically grown seed culture controls. This novel strategy might be a promising alternative to the current approaches to advance the production technology of astaxanthin from microalgae.

## Introduction

Astaxanthin (AX), a naturally occurring pigment from the xanthophyll group, is a high-value keto-carotenoid found in various microorganisms and marine animals (Ambati et al., 2014; Shang et al., 2016). AX is known as one of the most powerful natural antioxidants due to its exceptional antioxidant activity. It has been shown that astaxanthin has 500 times and 38 times higher antioxidant activity compared to vitamin E and β-carotene, respectively (Han et al., 2019). AX has been proven to have anti-inflammatory, immunomodulatory, anticancer, and antidiabetic effects (Niizawa et al., 2018; Han et al., 2019) and is well thought out to be beneficial for eye, joint, prostate, liver, and heart health, mostly due to its antioxidative property (Pan-utai et al., 2017). In addition, AX is an essential nutrient for the health and pigmentation of some farmed aquatic animals (Lim et al., 2018). Therefore, AX has a wide range of applications in the nutraceutical, cosmetic, and feed sectors of the industry (Hong et al., 2016; Wang et al., 2016; Zhao et al., 2019). The total market value of AX is estimated to reach US$800 million approximately by 2022 with 8% of compound annual growth rate (Li et al., 2020).

AX can be either chemically synthesized or biologically produced. Owing to its safety and stronger antioxidant potential, biological AX is preferred over chemically synthesized especially for human consumption. Among the sources of biological AX, the unicellular freshwater microalga *Haematococcus lacustris* is regarded as the richest natural source and a major producing organism of commercial AX. *H. lacustris* is capable of producing, primarily, the most valuable stereoisomeric form (3S, 3′S) of AX (Shah et al., 2016). *H. lacustris* has pretty complicated life cycles and morphologies. On the one hand, when the conditions are favorable (availability of nutrients, optimum light, and temperature) for growth, the cells remain in a green vegetative stage and actively multiply in the medium. On the other hand, when exposed to unfavorable (stressed) conditions, such as high light, nutrient deficiency (usually nitrogen), high temperature, and increased salinity, the cells undergo a dramatic transformation to red nonmotile hematocysts and completely stop cell division, developing thick cell walls and a high level of AX accumulated in the cytosol (Borowitzka, 2018). The massive accumulation of AX is mostly limited to the hematocyst stage under stressed conditions. Due to the biological factors, the two-step procedure characterized by biomass growth and AX production steps has been extensively employed for AX production from *H. lacustris* (Xi et al., 2016; Niizawa et al., 2018). For the two-step process, first, *H. lacustris* is cultivated typically in photobioreactors (PBRs) under a favorable environment for cell growth and biomass accumulation, and then cells are transferred to either larger-scale PBRs or open raceway ponds (ORPs) under unfavorable cultivation conditions to induce AX accumulation. The biomass production is usually performed in PBRs, and the AX accumulation stage is then performed in ORPs or outdoor tubular PBRs (Li et al., 2020).

Though after decades of technical development, the production costs of astaxanthin from *Haematococcus* is still high and is much higher than that of chemical astaxanthin, and thus, numerous innovations in AX production approaches such as two-stage photoautotrophic and heterotrophic cultivation stages followed by phototrophic and two-stage mixotrophic and attached cultivation have been reported (Park et al., 2014; Zhang et al., 2014; Shah et al., 2016). Due to the slow growth rates of *Haematococcus*, both the stages of cultivation are considered to be lengthy processes and frequently lead to low biomass and AX accumulation (Zhang et al., 2020), and ameliorating either process would improve the process economics. Inspired by the above ratiocination, a sequential heterotrophic and phototrophic cultivation strategy was proposed and tested (Hata et al., 2001; Wan et al., 2015). Although the heterotrophic cultivation of *Haematococcus* seemed pretty good, there might be several problems associated with this strategy. First, substantial amounts of cells could not survive the shifting from heterotrophic to phototrophic cultivation (Hata et al., 2001; Li et al., 2020). In order to minimize cell death when transferring, an additional step of acclimatization under moderate illumination was required prior to light induction (Zhang et al., 2016). Second, the heterotrophically cultivated cells seemed to lose, at least, partially the ability of photosynthesis in the phototrophic cultivation stage (Fang et al., 2019; Li et al., 2020). This phenomenon would greatly undermine the process efficiency of the astaxanthin accumulation stage. Similarly, the mixotrophic cultivation approach has long been considered as an alternate for traditional methods, and in fact, the highest AX accumulation was demonstrated by a mixotrophic cultivation approach (Park et al., 2014). However, the major drawbacks of this strategy are high process equipment and operational costs (Panis and Carreon, 2016). In addition, the application of this technique in large-scale ORPs is restricted because of bacterial contamination (Wen et al., 2020). In cultivations where organic carbon sources are supplied, bacteria and/or fungi find beneficial conditions to develop. Contamination could be a serious problem as process productivity and efficiency are reduced (Pleissner et al., 2020). Generally, bacterial contamination is unavoidable in outdoor microalgal cultivation and is intensified if organic carbon is available in the medium (Wen et al., 2019). Recently, a sequential mixotrophy dilution photoinduction strategy has been developed to boost the accumulation of astaxanthin in *Haematococcus* (Azizi et al., 2019). Although the alternative carbon sources were adopted to replace acetate, and the dilution procedure could reduce the risk of contamination, restricted sterile conditions might have to be maintained at the photoinduction stage. In order to improve the abovementioned methodologies, we proposed and developed a novel two-stage cultivation strategy, characterized by sequential continuous mixotrophic cultivation in indoor PBR systems for biomass growth and outdoor phototrophic cultivation in outdoor ORPs for astaxanthin accumulation. The developed strategy, which can be termed as sequential indoor continuous mixotrophic and outdoor phototrophic cultivation, might be able to overcome the drawbacks of both heterotrophic–phototrophic and mixotrophic–mixotrophic methods and thus might have the potential to improve the process economics of astaxanthin production from *H. lacustris.*

## Materials and Methods

### Strain and Media

The axenic freshwater microalgal *H*. *lacustris* strain 721*,* obtained from The Freshwater Algae Culture Collection at the Institute of Hydrobiology, Chinese Academy of Sciences (FACHB-collection), was used in this research. The strain was maintained in Bold’s Basal medium (BBM) agar plates (Nichols and Bold, 1965) under constant illumination of daylight LED (30 μmol photons m^−2^ s^−1^) at 22 ± 2°C. The sterility of cell cultures was monitored by microscopic examination (SGO-PH300, Kwong Kuk, China) and spreading the cultures on nutrient agar plates. To prepare the inoculum, the cells were transferred to 100 ml of sterilized BBM in 250 ml flasks and incubated at 22 ± 2°C, under constant illumination of daylight LED (50 μmol photons m^−2^ s^−1^). Cells in the exponential growth phase were used as the inoculum (10% v/v) for further indoor cultivation experiments.

### Indoor Cultivation System and Experimental Conditions

To determine optimal culture conditions for continuous mixotrophic cultivation, experiments were first performed in indoor closed PBR systems each composed of a 2 L bottle (Borosil® reagent bottle, 26.4 cm in height and 13.7 cm in diameter) with three ports for fresh medium supplement, biomass harvesting, and air blowing. The fresh medium was pumped into the PBRs using peristaltic pumps (Alledosieren, China) at varying dilution rates ranging from 0.18 to 0.72 days^−1^, and the culture flew out through the harvesting ports of the PBRs. The temperature of the PBRs was controlled at 23°C by placing the systems in a water bath connected to a cooling system. A 3% (v/v) CO_2_ and pressurized air stream was bubbled into the reactors through a sterile filter (0.22 µm) at a flow rate of 250 ml min^−1^. The PBRs were continuously illuminated by a daylight LED panel with the light intensity varying from 40 to 160 μmol photons m^−2^ s^−1^. The light photon flux density was measured using a series of quantum sensors (SQ-520, Apogee Instruments Inc., United States). Sodium acetate (NaAc) at a concentration of 5 mM was used as a carbon source in the feeding stream of BBM for mixotrophic cultivation. The microalgal culture was then scaled up to 10 L PBRs (41.7 cm in height and 23 cm in diameter) under optimized conditions using a similar setup as that of 2 L PBRs, and the resulting biomass was used as the seed culture for outdoor ORP cultivation experiments.

The batch phototrophic, heterotrophic, and mixotrophic experiments were performed in 2 L PBRs at a temperature of 23°C. The phototrophic and mixotrophic cultures were continuously illuminated by a daylight LED panel at a light intensity of 120 μmol photons m^−2^ s^−1^, while, for heterotrophic cultures, the light was avoided by covering the PBRs with an aluminum foil. 5 mM of Ac was used in heterotrophic and mixotrophic cultural experiments.

### Outdoor Open Raceway Ponds Cultivation System and Experimental Conditions

The outdoor cultivation of *H. lacustris* was carried out at Panzhihua University campus, Sichuan Province, China (with a latitude of 26°05′–27°21′ N and a longitude of 101°08′–102°15′ E) in ORPs from December 2020 to May 2021. The ORPs were made of polyvinyl chloride (PVC), each with dimensions of 2.0 m in length, 0.76 m in width, and 0.25 m in depth, and having a maximum working volume of 200 L with a water depth of roughly 0.15 m. To prepare pond media, 40 g NaHCO_3,_ 400 g NaCl, and 160 L tap water were added into the ponds. The cultivations were started by inoculating 40 L cell cultures grown in modified BBM with different levels of nitrogen (1.5-, 3-, and 5-fold) and containing 5 mM Ac (phototrophically grown seed cultures without Ac in the medium were cultivated and used as controls). The initial biomass concentrations of the seed cultures were maintained at 0.10 ± 0.01 g L^−1^. No additional nutrients or Ac was added to ORPs during the outdoor phototrophic cultivation period. Hereafter, the ORPs inoculated with mixotrophic seed cultures grown in 1.5-, 3-, and 5-fold nitrogen were denoted as ORP-1, ORP-2, and ORP-3, respectively, while the phototrophic seed cultures (controls) were ORPC_1_, ORPC_2_, and ORPC_3_. The pH of the medium was maintained at 8.0 by dispersing CO_2_ on demand by pH controllers. The cultures were continuously agitated at 20 rpm by eight-blade paddle wheels (0.64 m in diameter). Freshwater was added to the ponds daily to compensate for evaporation losses. All the experiments were performed in duplicate. The samples were collected daily and analyzed for biomass concentration, nitrate concentration, maximum efficiency of PSII photochemistry (*F*_v_/*F*_m_ ratio), and pigment content determination.

### Biomass and Pigment Measurement

To determine the biomass in terms of dry cell weight (DCW), 50 ml of samples were filtered through predried and preweighted glass fiber filter membranes (Shanghai Xingya, China), and then, the membranes were washed twice with deionized water before being dried at 70°C to a constant weight. The biomass DCW was calculated in g L^−1^, and the biomass productivity (g L^−1^ d^−1^) was calculated by dividing the amount of biomass per liter volume and the duration of cultivation. In continuous modes, the biomass productivity (g L^−1^ d^−1^) was calculated as the product of dilution rates (day^−1^) and the steady-state biomass concentrations (g L^−1^).

Pigments were quantified according to the method described by Wen et al. (2020). Briefly, the culture suspension was centrifuged to collect the cells, followed by washing with deionized water. The pigments were then extracted twice using dimethyl sulfoxide (DMSO) at 70°C for 10 min. The extract was centrifuged, and the absorbance of the supernatant was measured at 480 nm, 530 nm, 650 nm, and 666 nm. The total contents of carotenoids, astaxanthin, and chlorophyll a and b were calculated, using the equations described by Wen et al. (2020).

### Determination of Nitrogen (NO_3_-N) and Acetate

The culture suspension was centrifuged, and the supernatant was then filtered through a 0.2 µm syringe filter. The filtrates were used to measure NO_3_-N and acetate concentrations. NitraVer® 5 Nitrate Reagent Powder Pillows (Hach) were used to determine NO_3_-N concentrations, and Megazyme acetic acid kits (K-ACETRM) were used to determine acetate levels. The experiments were performed according to the manufacturer’s instructions.

### Photosystem II Maximum Quantum Yield Measurement

The maximum photochemical quantum yield (*F*_*v*_ */F*_*m*_) of the photosystem II was determined by a pulse-amplitude-modulated (PAM) fluorometer (AquaPen-C, AP 110-C, Photon Systems Instruments, Czech Republic) with a blue light source (455 nm). The cell suspension culture was first adapted in the dark for 15 min. The dark-adapted samples (3 ml) were then applied for measuring cuvettes (a light path of 10 mm). The dark-adapted minimum levels of fluorescence (*F*_*0*_) and the maximum levels of fluorescence (*F*_*m*_) were measured before and after a short saturating light pulse (3,000 µmol photons m^−2^ s^−1^ with a duration time of 0.6–0.8 s). The photon flux density of excitation light was 0.09 μmol photons m^−2^ s^−1^, and fluorescent light was detected in a spectral range of 667–750 nm. The *F*_*v*_ */F*_*m*_ value was calculated as *F*_*v*_ */F*_*m*_ = (*F*_*m*_ − *F*_*0*_)/*F*_*m*_ (Maxwell and Johnson, 2000).

### Bacterial Cell Count

The bacterial cell number in the ORPs was determined by colony-forming unit (cfu) counting. The cell culture samples were first serially diluted and then spread on nutrient agar plates. The nutrient agar medium was composed of 5 g L^−1^peptone, 3 g L^−1^ beef extract, 5 g L^−1^sodium chloride, and 15 g L^−1^ agar. The plates were incubated at 37°C for 24–48 h, and the plates with colony numbers between 30 and 300 were used to count the cfu (Wen et al., 2019).

### Statistical Analysis

All experiments were conducted in duplicate. The average values and standard deviations (SD) were calculated, and the results were shown in mean ± SD.

## Results and Discussion

### Mixotrophic Cultivation Was Superior to Phototrophic Cultivation for Green Cell Biomass Growth

**FIGURE 1:**
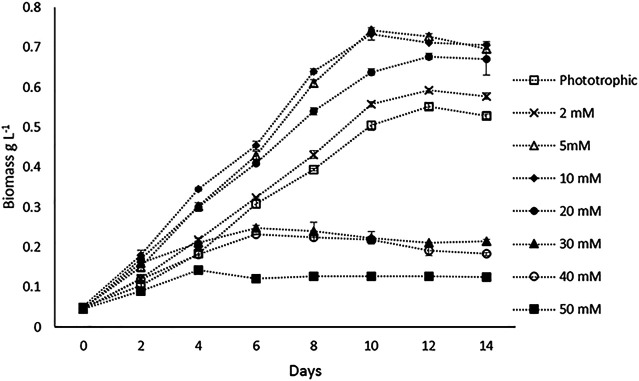
Biomass (DCW) profiles of *H. lacustris* under phototrophic and mixotrophic cultivation in the batch mode.

Batch experiments were performed to compare the biomass productivity of *H. lacustris* under phototrophic and mixotrophic cultivations. Prior to batch studies, Ac concentrations were optimized for mixotrophic cultivation. The concentrations of the Ac tested were 2, 5, 10, 20, 30, 40, and 50 mM, and optimal growth was observed at 5 and 10 mM. At both concentrations, there was no significant difference in the maximum DCW observed (Figure 1). Growth rates were decreased, with the concentrations getting higher than 10 mM. Thus, in batch experiments 5 mM Ac was used in mixotrophic cultures. The DCW profiles of the batch cultivations of both phototrophic and mixotrophic modes and the influences of Ac concentrations on biomass are shown in Figure 1. Previous studies have reported varied results regarding the optimum concentrations of Ac for the mixotrophic cultivation of *H. lacustris*. For instance, 6.2 mM NaAc was found to be optimal for *H. lacustris* in the study conducted by Gong and Chen (1997), whereas Goksan and Gokpinar (2010) obtained the best results at 12 mM NaAc. On the other hand, higher concentrations of NaAc such as 30 mM (Jeon et al., 2006) and 50 mM (Pan-utai et al., 2017) were also found to be optimal for *H. lacustris.* Different strains of *H. lacustris* might respond differently to Ac for their optimal growth, and other operational conditions might also contribute to the overall outcomes. Although it is noteworthy that acetate at higher concentrations could be toxic to the cells and adversely affect their growth (De Swaaf et al., 2003), in the presence of low concentrations of Ac, various microalgae can grow well. Various studies also reported the utilization of Ac to not only enhance the growth of green cells but also induce the encystment of *H. lacustris* (Cifuentes et al., 2003; Cong et al., 2020). In the present study, microscopic observations showed that cells did not form cysts when 5 mM Ac was added into the medium in the initial days of cultivation for batch experiments. However, as cultivation prolonged (after 12 days), the cysts were observed, which might be due to the depletion of nutrients in the culture medium. Various other studies have also reported the activation of cyst formation in the presence of Ac and high light irradiance for astaxanthin production (Domınguez-Bocanegra et al., 2004; Jeon et al., 2006).

Our experimental results showed that *H. lacustris* cultivated under mixotrophic conditions grew faster and resulted in higher DCW (0.743 g L^−1^) and biomass productivity (0.071 g L^−1^ d^−1^), compared to phototrophic cultures (DCW; 0.556 g L^−1^ and biomass productivity; 0.049 g L^−1^ d^−1^) (Figure 1). Considering the fact that the addition of exogenous organic carbon provided both carbon sources and chemical energy for mixotrophic cultivation, we were not surprised to observe a significant improvement in biomass productivity, compared to phototrophic cultures with the same levels of light supply. The results are in agreement with earlier reports that the growth rates of *H. lacustris* under mixotrophic conditions supplemented with Ac and other carbon sources were much quicker than those of photoautotrophic cultures (Goksan and Gokpinar, 2010; Pang and Chen, 2017; Wen et al., 2020). Indoor cultivation was instrumental in eliminating the risk of contamination since aseptic operations could be easily applied, and it was not challenging to achieve high cell concentrations in closed systems, with Ac as the carbon source under sterile mixotrophic conditions (Chojnacka and Noworyta, 2004).

### Continuous Cell Culture Was More Productive Than Batch Cell Culture for *H. lacustris* Cultivation

For cost-effective astaxanthin production from *H. lacustris*, boosted biomass productivity in the green phase is highly essential (Li et al., 2020). Continuous mixotrophic cultivation of *H. lacustris* was performed in parallel, with Ac as a carbon source, to enhance biomass productivity. Continuous phototropic cultivation was also performed in parallel to mixotrophy in order to obtain data for comparison. Light intensity and the dilution rate were considered as major influencing factors for continuous processes. Thus, different light intensities (40, 80, 120, and 160 μmol photons m^−2^ s^−1^) and dilution rates (0.18, 0.36, 0.54, and 0.72 days^−1^) were tested for both phototrophic and mixotrophic cultures to optimize the cell growth. Steady states were attained under all dilution rates and light intensities. The average values of 5-day steady-state biomass concentration, productivity, and chlorophyll content are shown in Table 1. As indicated by the results, the significant effects of dilution rates on biomass concentrations were observed. The steady-state biomass concentrations in both phototrophic and mixotrophic cultures were gradually increased with decreased dilution rates under all light intensities (Table 1). A maximum steady-state biomass concentration of 0.402 ± 013 gL^−1^ was attained at the dilution rate of 0.18 days^−1^ under the light intensity of 160 μmol photons m^−2^ s^−1^ in mixotrophic cultures. This value is 1.5 times higher than that obtained in phototrophic cultures under the same conditions (Table 1). In continuous cultivation, the longer the residence time, the higher the substrate utilization, and thus, the cell biomass concentrations are higher at lower dilution rates than those at higher dilution rates (Liu, 2013). Under steady-state cultivation, the dilution rates of continuous cell cultures are, in fact, equivalent to the cell growth rates (López-Hernández et al., 2020). However, the maximum biomass productivity of 0.104 ± 003 g L^−1^ d^−1^ was attained at a dilution rate of 0.54 days^−1^ under a light intensity of 160 μmol photons m^−2^ s^−1^ in mixotrophic cultures. In the case of phototropic cultures, the biomass productivity was 0.060 ± 001 g L^−1^ d^−1^ under the same conditions. A further increase in the dilution rate from 0.54 to 0.72 days^−1^ did not show any increment in biomass productivity, due to reduced biomass concentrations. Therefore, the dilution rate of 0.54 days^−1^ and the light intensity of 160 μmol photons m^−2^ s^−1^ were considered as optimal to attain the highest biomass productivity in our experimental conditions.

**TABLE 1:** Steady-state biomass (DCW), chlorophyll content, and biomass productivity.

Irradiance (μmol photons m^−2^ s^−1^) and dilution rate (d^−1^)		Phototrophic			Mixotrophic		
		Steady-state biomass concentration (g L^−1^)	Steady-state chlorophyll content (% DW)	Biomass productivity (g L^−1^ d^−1^)	Steady-state biomass concentration (g L^−1^)	Steady-state chlorophyll content (%DW)	Biomass productivity (g L^−1^ d^−1^)
40	0.18	0.127 ± 002	2.832 ± 0.094	0.022 ± 000	0.205 ± 004	2.232 ± 0.031	0.037 ± 002
	0.36	0.10 ± 002	2.878 ± 0.053	0.036 ± 001	0.152 ± 002	2.258 ± 0.052	0.054 ± 001
	0.54	0.075 ± 002	3.086 ± 0.071	0.040 ± 000	0.118 ± 003	2.522 ± 0.034	0.063 ± 001
	0.72	0.052 ± 001	2.812 ± 0.040	0.037 ± 001	0.081 ± 002	2.072 ± 0.053	0.058 ± 001
80	0.18	0.216 ± 004	2.808 ± 0.078	0.038 ± 000	0.311 ± 005	2.208 ± 0.034	0.056 ± 002
	0.36	0.123 ± 003	2.908 ± 0.068	0.044 ± 001	0.166 ± 004	2.228 ± 0.039	0.059 ± 001
	0.54	0.091 ± 002	3.104 ± 0.062	0.049 ± 001	0.128 ± 002	2.532 ± 0.010	0.069 ± 003
	0.72	0.064 ± 002	2.78 ± 0.055	0.046 ± 002	0.089 ± 001	2.08 ± 0.055	0.064 ± 003
120	0.18	0.248 ± 002	2.61 ± 0.074	0.044 ± 002	0.355 ± 004	2.09 ± 0.070	0.063 ± 002
	0.36	0.130 ± 001	2.634 ± 0.057	0.047 ± 001	0.193 ± 003	2.168 ± 0.032	0.069 ± 002
	0.54	0.095 ± 002	3.108 ± 0.073	0.051 ± 001	0.144 ± 002	2.576 ± 0.050	0.078 ± 002
	0.72	0.068 ± 001	2.628 ± 0.016	0.048 ± 002	0.101 ± 003	2.048 ± 0.099	0.073 ± 003
160	0.18	0.261 ± 008	2.412 ± 0.043	0.047 ± 001	0.402 ± 013	1.992 ± 0.050	0.072 ± 001
	0.36	0.145 ± 003	2.414 ± 0.049	0.052 ± 001	0.224 ± 004	2.034 ± 0.069	0.080 ± 002
	0.54	0.112 ± 002	2.446 ± 0.081	0.060 ± 001	0.194 ± 005	2.186 ± 0.058	0.104 ± 003
	0.72	0.076 ± 001	2.354 ± 0.051	0.055 ± 002	0.122 ± 002	1.982 ± 0.080	0.087 ± 004

As shown in Table 1, continuous mixotrophic cultivation showed better productivity (0.104 ± 003 g L^−1^ d^−1^), compared to the continuous phototrophic (0.060 ± 001 g L^−1^ d^−1^), batch mixotrophic (0.071 g L^−1^ d^−1^), and batch phototrophic cultivation (0.049 g L^−1^ d^−1^). The productivity achieved in the present study is much higher than industrial-scale productivity, which employed a semicontinuous phototrophic approach (Olaizola, 2000). Therefore, it can be considered that continuous mixotrophic cell culture could be much more productive than batch and phototrophic continuous culture for *H. lacustris* cultivation. The batch culture of *H. lacustris* commonly suffers nutrient deficiency during the later stages of cultivation, leading to cyst formation and inhibition of cell proliferation (Choi et al., 2011; Niizawa et al., 2018). Here, in continuous cultures, a constant nutrient supply was maintained, which might be another reason for better performance. The photosynthetic efficiency of the cells under continuous cultivation was higher than that of batch cultures. The measured *F*_v_/*F*_m_ values of both phototrophic and mixotrophic continuous cultures were around 0.7 throughout the cultivation period, and the photosynthetic efficiency was not affected by various levels of nitrogen in our experimental settings. Previously, Pang et al. (2019) had reported that the mixotrophic cells of *Haematococcus* also showed a healthy status and highly efficient PSII activity. Although the cells under mixotrophic conditions showed decreased *F*_*v*_ */F*_*m*_ values for the first 2 days, it seemed that the photosynthetic ability of cells recovered after 5 days of cultivation. On the contrary, Zhang et al. (2019) reported a significant decrease in *F*_v_/*F*_m_ values in Ac-treated cells, compared with cells without Ac. In another study, the *F*_*v*_ */F*_*m*_ values of vegetative *Haematococcus* cells in the exponentially growing phase were increased from 0.46 ± 0.01 to 0.50 ± 0.01 during the first 3 days of the cultivation, clearly showing the upregulation of photosynthesis (Chekanov et al., 2017). It was observed that the amplitude of the maximum chlorophyll fluorescence peak of *Haematococcus* had declined considerably under stressed conditions, while in the course of vegetative growth recovery, the maximum photosystem II quantum yield returned to the levels characteristic of the vegetative cells within 80 h (Chekanov et al., 2016).

Our experimental results showed that by using continuous mixotrophic cultivation, high biomass productivity could be achieved continuously for prolonged periods, and high photosynthesis efficiency could be maintained. To the best of our knowledge, this study was the first attempt in optimizing the continuous mixotrophic cultivation of *H. lacustris*. Previously, the one-step continuous photoautotrophic cultivation of *H. lacustris* had been performed under moderate nitrogen levels for simultaneous cell growth and astaxanthin accumulation (Río et al., 2005; Del Río et al., 2008). Later, the feasibility of this continuous cultivation method was also tested in outdoor tubular PBRs with a specific amount of nitrate input (García-Malea et al., 2009).

### Mixotrophically Cultivated *Haematococcus* Cells Were Still Be Able to Maintain High Levels of Photosynthetic Capacity

The chlorophyll contents of *Haematococcus* cells were measured under batch cultivations of phototrophic, heterotrophic, and mixotrophic cultures to compare the photosynthetic ability of cells under these three modes. Results showed that total chlorophyll contents (chlorophyll a and b) were higher under phototrophic (40.1 mg g^−1^) conditions than those under heterotrophic (9.5 mg g^−1^) and mixotrophic (34.6 mg g^−1^) conditions. In heterotrophic cultures, the decrease of chlorophyll contents was even visible for the color of cells which changed to light yellow (data not shown). In mixotrophic cultures, the chlorophyll contents were lower than those in phototrophic cultures but much higher than those in heterotrophic cultures. It was confirmed that mixotrophic cells maintained much higher levels of photosynthetic ability than heterotrophic cells. Chlorophyll contents in mixotrophic cultures were significantly higher and actually at levels comparable to those in the phototrophic cultures. As expected, the maximum photosynthetic ability of cells was observed in phototrophic modes and followed by that in mixotrophic cultures, and the least amount of chlorophyll contents was found in heterotrophic cell cultures. Previously, the photosynthetic ability of mixotrophically grown microalgae had been found close to phototrophic cultures (Xie et al., 2016), although nearly 50% reduction of the chlorophyll content in mixotrophy was also reported (Cecchin et al., 2018).

**FIGURE 2:**
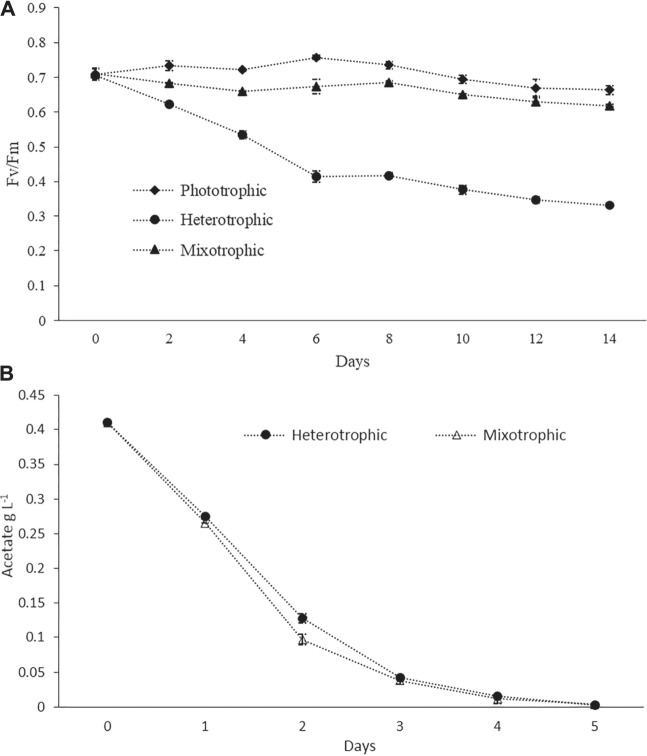
**(A)** Photosynthetic efficiency measured as PSII quantum yield (*F*_v_/*F*_m_) in phototrophic, heterotrophic, and mixotrophic cultures. **(B)** Ac consumption profiles in the batch mode.

Pigment–protein complexes related to PSII reaction centers were known to be damaged by inorganic nitrogen depletion, which leads to a decrease in the biomass yield and the *F*_v_/*F*_m_ values (Remmers et al., 2017). For healthy microalgal cells, *F*_v_/*F*_m_ values are expected to be around 0.6–0.7, while lower values are an indication of stressed conditions (Young and Beardall, 2003;Benvenuti et al., 2015). In the present study, the *F*_v_/*F*_m_ values in phototrophic batch cultures always remained higher than 0.7 during the logarithmic growth phase, but a slight decrease was observed toward the end of the cultivation period (Figure 2A) which may be due to nitrogen depletion. In heterotrophic cultures, the value decreased continuously during the cultivation up to values lower than 0.4 (Figure 2A). This could be attributed to the disassembly of photosynthetic complexes under heterotrophic conditions. Our results were in agreement with the previous study where a sharp decrease in the Fv/Fm value had been reported for heterotrophically grown cells (Zhang et al., 2016). In the case of mixotrophic cultures, the values were slightly decreased during the initial 4 days, then gradually increased from day 5 up to day 10, and again decreased after day 10 (Figure 2A). This pattern might indicate that in the initial days and due to the availability of Ac, the photosynthetic efficiency was low, and after the consumption of Ac, the cells shifted to active photosynthesis. The Ac consumption profiles under batch cultivation are given in Figure 2B. Similarly, at the late stage of cultivations and due to the damage of photosystem reaction centers under nutrient-depleted conditions (especially nitrogen), the values were decreasing (Remmers et al., 2017). However, the values were always higher than 0.6 throughout the entire cultivation period (Figure 2A). A recent study used *F*_v_/*F*_m_ values as an indicator of the wellness of the photosynthetic apparatus. It has reported a similar finding that *F*_v_/*F*_m_ values of both phototrophic and mixotrophic cultures were around 0.6 throughout the entire cultivation duration (Cecchin et al., 2018). Worthy of mentioning is that, in a continuous culture study, *F*_v_/*F*_m_ ratios were found to be greater than 0.6 under nitrogen starvation, but the photosynthetic rates were significantly decreased. This fact indicated that the *F*_v_/*F*_m_ values might not always be a perfect indicator of stresses, and various other factors in the photosystem could also affect photosynthetic efficiency, which were not reflected by the *F*_v_/*F*_m_ values (Remmers et al., 2017).

### Phototrophic Cultivation in Outdoor Raceway Ponds Was Efficient for *H. lacustris* to Accumulate Astaxanthin With Mixotrophically Cultivated Seed Culture

**FIGURE 3:**
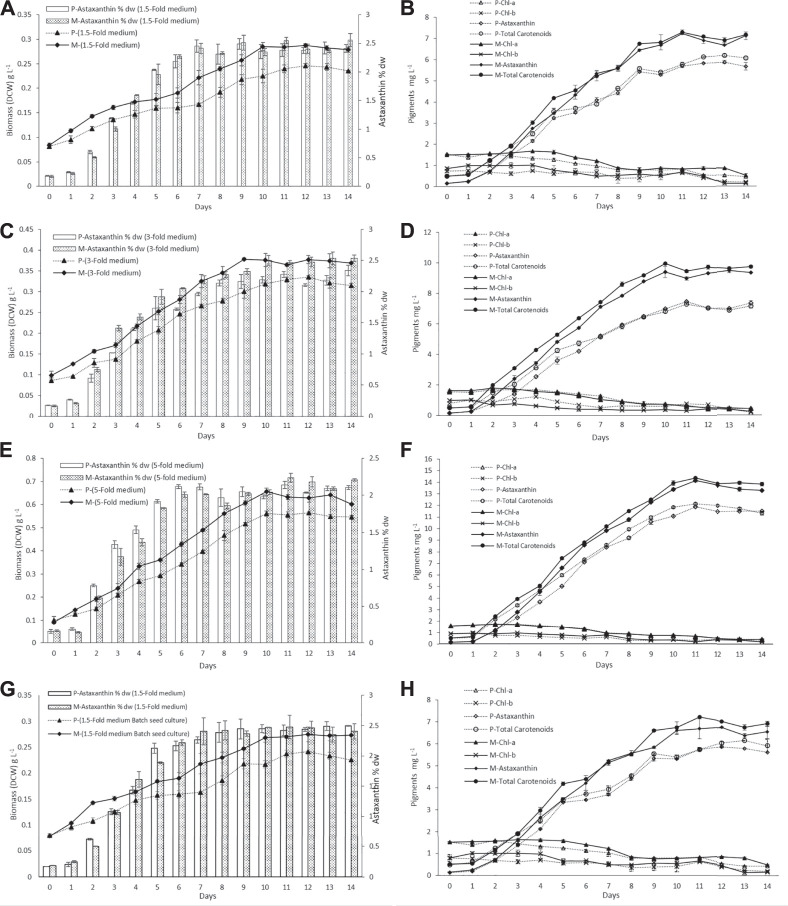
Dry cell weight and pigment profiles of *H. lacustris* grown in outdoor ORPs, with phototrophically grown seed cultures (labeled with P) and mixotrophically grown seed cultures (labeled with M). **(A)** Biomass DCW and astaxanthin content % DW profiles in 1.5-fold medium; **(B)** pigment profiles in 1.5-fold medium; **(C)** biomass DCW and astaxanthin content % DW profiles in 3-fold medium; **(D)** pigment profiles in 3-fold medium; **(E)** biomass DCW and astaxanthin content % DW profiles in 5-fold medium; **(F)** pigment profiles in 5-fold medium; **(G)** biomass DCW and astaxanthin content % DW profiles of batch cultivation seed cultures; **(H)** pigments profile of batch cultivation seed culture.

The biomass DCW and pigment profiles of *H. lacustris* grown in outdoor ORPs are depicted in Figure 3. The results showed that the culture medium had significant effects on cell growth and biomass concentrations during the photoinduction period for both phototrophic and mixotrophic seed cultures. Seed cultures prepared in a 5-fold nitrogen medium (ORP-3) had shown significant improvements compared to 1.5-fold (ORP-1) and 3-fold (ORP-2). Maximum biomass concentration (0.655 g L^−1^) was obtained in ORP-3 with the mixotrophic seed culture grown in the 5-fold nitrogen medium. This value was 1.1 times higher than the phototrophic 5-fold seed culture (ORP-C_3_) and 2.2 times higher than that obtained in the 1.5-fold mixotrophic seed culture (ORP-1) (Figures 3A,C,E). However, further increase in nutrients (7.5-fold nitrogen BBM) in seed cultures did not show significant improvements in biomass concentrations (data not shown). Therefore, 5-fold BBM might be optimal to achieve high cell density in the outdoor photoinduction period. In cases of batch cultivation seed cultures, the highest biomass concentration and astaxanthin content were 0.275 g L^−1^ and 2.42% DW, respectively (Figures 3G,H).

Previously, it had been reported that heterotrophically grown cultures were unable to cope with high irradiance when subjected to phototrophic cultivation and resulted in a substantial amount of cell death. To minimize the cell death of heterotrophically grown seed cultures, an additional acclimatization step was required before induction (Hata et al., 2001; Zhang et al., 2016). In the present study, the mixotrophically grown cultures were directly subjected to outdoor phototrophic cultivation, and cell death was not observed. Chlorophyll a and b contents of the cultures (both phototrophic and mixotrophic seeds) slightly increased during the initial 4–5 days while cells were growing, and thereafter, the chlorophyll levels were decreasing during the formation of aplanospores (hematocysts). Chlorophyll a and b contents of all test ORPs along with the control ORPs are illustrated in Figures 3B,D,F,H. The decrease in chlorophyll contents at later stages could be attributed to a significant decrease in the expression of chlorophyll biosynthesis genes during astaxanthin accumulation and the inhibition of chlorophyll biosynthesis under nitrogen-depleted conditions (Scibilia et al., 2015; Zhang et al., 2019; Zhao et al., 2019). Similar to the present results, previous studies had reported a decrease in chlorophyll content during the astaxanthin accumulation stage (Fábregas et al., 2001; Zhang et al., 2019).

**FIGURE 4:**
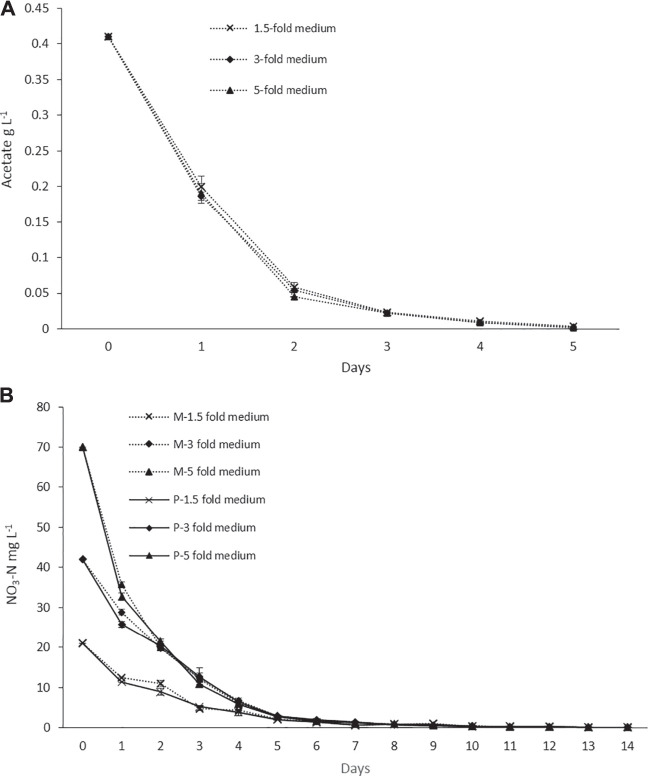
**(A)** Acetate consumption profiles of ORP cultivation with mixotrophic seed cultures. **(B)** Nitrogen consumption profiles of ORP phototrophic (labeled with P) and mixotrophic (labeled with M) cultures.

The nitrogen and acetate consumption profiles of ORPs are shown in Figures 4A,B. According to the tests, both Ac and nitrogen consumption rates were high during the early stage of the photoinduction period until the 5^th^ day when they were completely assimilated (Figures 4A,B). Under nitrogen-depleted conditions, intracellular nitrogen reserves were reported to support the cell growth for several days (Wen et al., 2020), while in mixotrophic cultures, upon the depletion of Ac, the cells, in fact, grew phototrophically (Goksan and Gokpinar, 2010). According to our observations, the cells were in a green vegetative phase during the initial 2 days of the photoinduction period, whereas, after the depletion of nutrients and being subjected to high light conditions, the cultures gradually changed to reddish cysts. Astaxanthin accumulation was progressively increased from day 2 to day 10. Furthermore, the astaxanthin contents in mixotrophic seed ponds were higher than those in control (phototrophic seed) ponds. The maximum astaxanthin content observed in ORP-1, ORP-2, and ORP-3 were 7.23 mg L^−1^ (2.5% DW), 9.47 mg L^−1^ (2.5% DW), and 14.14 mg L^−1^ (2.2% DW), respectively. However, in control ponds, the values were 5.8 mg L^−1^ (2.4% DW), 7.46 mg L^−1^ (2.2% DW), and 11.8 mg L^−1^ (2.1% DW), respectively, for ORPC_1_, ORPC_2_, and ORPC_3_ (Figures 3A,C,E). Taking together, phototrophic cultivation in outdoor raceway ponds was efficient for mixotrophically cultivated *H. lacustris* seed culture to accumulate astaxanthin because no cell death and loss of photosynthetic efficiency were observed. Additionally, mixotrophically cultivated *H. lacustris* seed cultures for outdoor photoinduction were advantageous to photosynthetic seed cultures since significantly increased cell biomass concentrations and astaxanthin contents were obtained. The residues of Ac in the seed culture medium were almost completely consumed by *H. lacustris* during the initial 2–3 days of the cultivation (Figure 4A), which essentially reduced the chances of bacterial contamination.

The mixotrophic cultivation of *H. lacustris* was mostly performed under laboratory conditions, and mixotrophy in ORPs is rather rare due to an unavoidable bacterial contamination problem outdoors. Therefore, in this study, mixotrophically grown cultures were cultivated phototrophically in ORPs. However, Ac was still present in the seed medium at relatively low concentrations. Thus, in order to compare the bacterial abundance in ponds with different seed cultures, the bacterial count has been estimated daily for 6 days, and the results are given in Table 2. The bacterial growth could positively correlate with the levels of readily accessible nutrients in the culture medium, and the deficiency of these nutrients can potentially limit bacterial growth (Wen et al., 2020). This was in accordance with our results which showed that the bacterial number was initially increased and then decreased a little bit till day 4 when it became almost constant. A similar trend was observed for both ORPs with phototrophic and mixotrophic seeds. The bacterial count number was higher in the mixotrophic seed pond (1.1 × 10^6^ cfu ml^−1^) than in the phototrophic seed pond (1.9 × 10^5^ cfu ml^−1^) at day 1. This might be due to the presence of Ac in mixotrophic seeds which resulted in a significant initial increase of bacterial counts. With the consumption of Ac, the nutrient availability in both mixotrophic and phototrophic seed ponds became similar, leading to comparable levels of bacterial counts. Bacterial contamination in the outdoor cultivation of *H. lacustris* was rarely investigated. A recent study investigating the daily dynamics of *H. lacustris* microbiome revealed interesting consortia between microalgae and bacteria. The findings provided an insight into possible bacterial contamination of the harvested algal biomass and also revealed the possible presence of a core microbiome which may be essential for the growth of the microalga although an interesting question whether there was a core *H. lacustris* microbiome still needed further confirmation (Chekanov et al., 2021). In another recent study, Wen et al. (2020) performed mass cultivation of *H. lacustris* in ORPs under the mixotrophic mode. The cells were first cultivated phototrophically, and then acetate was added under nitrate-depleted conditions to limit the bacterial contamination in ORPs. It was reported that the average abundance of bacteria was only slightly higher in mixotrophic cultures (8.9 × 10^5^ cfu ml^−1^), compared to phototrophic cultures (2.8 × 10^5^ cfu ml^−1^), while in the present study, the bacterial count was only found high at day 1 in mixotrophic seeds and was comparable to phototrophic seed for the rest of the cultivation period (Table 2). Taking together, it could be thought that mixotrophically grown cultures could be effectively used as seed culture in outdoor phototrophic cultivation since the risk of contamination is relatively low, compared to outdoor mixotrophy, and might be comparable to outdoor phototrophy, which was rather acceptable.

**TABLE 2:** Bacterial count in outdoor ORPs.

Days	Bacterial number CFU mL^−1^	
	Phototrophic seed ORP	Mixotrophic seed ORP
0	3.6 × 10^3^	4.1 × 10^3^
1	1.9 × 10^5^	1.1 × 10^6^
2	2.1 × 10^5^	3.8 × 10^6^
3	3.8 × 10^5^	4.5 × 10^6^
4	5.5 × 10^4^	9.6 × 10^4^
5	2.1 × 10^4^	4.4 × 10^4^
6	1.7 × 10^4^	4.1 × 10^4^

### Sequential Indoor Continuous Mixotrophic and Outdoor Phototrophic Cultivation in Raceway Ponds Might Be an Effective Cultivation Strategy to Substantially Reduce the Production Cost

**FIGURE 5:**
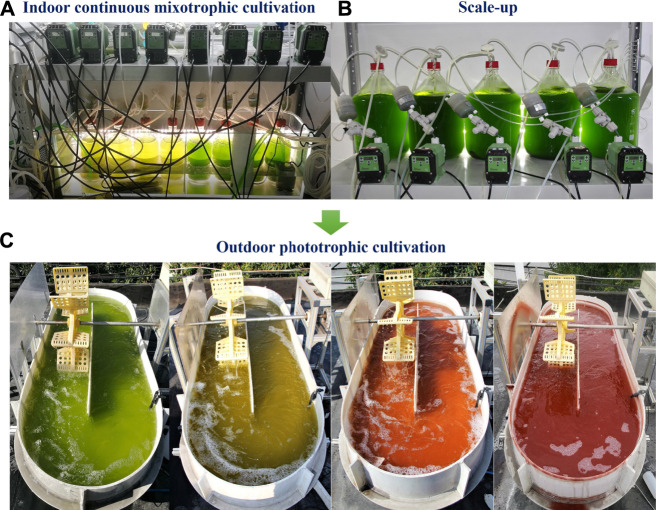
The process of sequential indoor continuous mixotrophic and outdoor phototrophic cultivation. **(A)** Indoor continuous mixotrophic cultivation of *H. lacustris* (BBM with 5 mM Ac) in 2 L PBRs carried out at a temperature of 23°C under a light intensity of 160 μmol photons m^−2^ s^−1^, 3% (v/v) CO_2_, and pressurized air bubbling at a flow rate of 250 ml min^−1^. **(B)** Scale-up of continuous mixotrophic cultivation in 10 L PBRs to prepare seed cultures of *H. lacustris* for ORPs (modified BBM with different levels of nitrogen, 5 mM Ac, at a dilution rate of 0.54 days^−1^ under a light intensity of 160 μmol photons m^−2^ s^−1^, 3% (v/v) CO_2_, and pressurized air bubbling at a flow rate of 250 ml min^−1^). **(C)** Outdoor phototrophic cultivation of *H. lacustris* in ORPs to produce astaxanthin (14-day cultivation period).

High production cost has been the biggest obstacle associated with astaxanthin production from microalgae, and novel cultivation strategies might have the potential to substantially reduce the cost. Two principles can be followed to develop novel strategies for cost reduction. One is to use inexpensive cultivation systems such as ORPs instead of expensive PBRs for large-scale cultivation because the major capital cost of the industrial production facility is from cultivation equipment. Another one is to improve the efficiency of seed culture supply since substantial amount of operational cost is from seed culture cultivation (Li et al., 2011; Li et al., 2020). In the present study, a novel cultivation strategy referred to as sequential indoor continuous mixotrophic and outdoor phototrophic cultivation (Figure 5) was proposed as a promising methodology to substantially reduce the production cost. First, this strategy enabled us to produce *Haematococcus* seed culture for astaxanthin production in ORPs in a more efficient manner than other indoor or even outdoor phototrophic methods. Indoor cultivation of *Haematococcus* in PBRs even with LED as the light source consumes extremely high amounts of electrical energy not only because of lighting but also cooling. Mixotrophic indoor cultivation could reduce substantial amounts of both lighting and cooling energy consumption. The continuous operation of the process could further improve the process efficiency and reduce the labor work of the assembly and disassembly of cultivation equipment. The outdoor cultivation of seed culture in PBRs with sunlight as the light source does not consume as much energy as indoor cultivation for lighting, but the temperature control of outdoor PBRs might be very challenging and costly. Back in the early 2000s, Olaizola (2000) reported a commercial case of the phototrophic cultivation of *Haematococcus* culture in outdoor PBRs semicontinuously as seed culture supply, but the reported location of the production facility was in Hawaii Ocean Science and Technology Park where inexpensive cold deep-sea water was conveniently available and the temperature control of large-scale outdoor PBRs could be easily managed (Olaizola, 2000; Olaizola, 2003). To reduce the high cost associated with seed culture supply, a sequential heterotrophic and phototrophic cultivation strategy was proposed and tested by Wan et al. (2015). However, the methodology had problems of high levels of cell death, low levels of photosynthetic efficiency, and the need for cell acclimatization upon transferring the seed culture to outdoor cultivation systems although the potential cost reduction on expensive large-scale PBRs might be achieved by using ORPs. To avoid the problems mentioned above, our proposed methodology replaces indoor heterotrophic cultivation with continuous indoor mixotrophic cultivation for seed culture supply for outdoor ORPs. Our experimental results showed that mixotrophic *Haematococcus* cultures could be directly used as seed cultures in outdoor phototrophic cultivation without acclimatization since their photosynthetic apparatus was not harmed. Mixotrophy performed under the continuous mode could further reduce the difficulties of assembly and disassembly of equipment that were associated with batch cultures. The quality of *Haematococcus* seed cultures was successfully justified by our outdoor experiments in ORPs, which were the major results of this research. Our strategy reduced the cost related to both the seed culture supply and the large-scale cultivation for AX production since inexpensive ORPs instead of expensive tubular glass PBRs could be used (Acién et al., 2017). Cell cultivation in ORPs often suffered from serious contamination because they are open systems, and only high-quality seed culture could be utilized for production in ORPs even at the photoinduction stage. The large-scale cell culture of *Haematococcus* in ORPs often encountered collapse due to the contaminated seed culture (Li et al., 2020). This might be the most important reason why expensive tubular glass PBRs were widely used for the large-scale cultivation of *Haematococcus* to produce astaxanthin. In the current study, indoor continuous mixotrophic cultivation under sterile conditions was proven to be able to ensure the quality of seed culture for outdoor cultivation in ORPs in a relatively less labor-intensive manner, and also this way, expensive outdoor PBR systems were avoided. Our experiments clearly demonstrated mixotrophic seed culture performed even better than phototrophic seed culture in ORPs, and the contamination of bacteria could be under control. Taking all the points together, we intended to claim that our proposed strategy might be a promising novel approach to reduce the cost of production of astaxanthin from microalgae.

## Conclusion

We proposed and developed a novel strategy for the large-scale cultivation of *Haematococcus* to produce astaxanthin, which we termed as sequential indoor continuous mixotrophic and outdoor phototrophic cultivation. The literature indicated that mixotrophy could be an effective strategy to cultivate *Haematococcus* especially for biomass accumulation, and our experiments clearly showed that higher productivity could be achieved by mixotrophic cultivation than phototrophic cultivation under indoor conditions with the same levels of light supply. At the same time, mixotrophically cultivated cells were proven to be able to grow phototrophically with high levels of photosynthetic efficiency and to maintain the high capacity of astaxanthin accumulation upon light induction. When mixotrophically cultivated *Haematococcus* cell cultures were diluted and inoculated in outdoor raceway ponds, satisfactory cell growth and astaxanthin accumulation yields were achieved. In conclusion, sequential indoor continuous mixotrophic and outdoor phototrophic cultivation might serve as a novel and promising strategy for the large-scale cultivation of *Haematococcus* to industrially produce biological astaxanthin.

## Data Availability

The original contributions presented in the study are included in the article/Supplementary Material; further inquiries can be directed to the corresponding author.
